# Quantitative Trait Loci Associated with Phenological Development, Low-Temperature Tolerance, Grain Quality, and Agronomic Characters in Wheat (*Triticum aestivum* L.)

**DOI:** 10.1371/journal.pone.0152185

**Published:** 2016-03-28

**Authors:** D. B. Fowler, A. N'Diaye, D. Laudencia-Chingcuanco, C. J. Pozniak

**Affiliations:** 1 Department of Plant Sciences and Crop Development Centre, University of Saskatchewan, Saskatoon, SK, Canada, S7N 5A8; 2 Crop Improvement and Genetics Research Unit, USDA-ARS WRRC, 800 Buchanan St. Albany, CA, United States of America, 94710; NSW Department of Primary Industries, AUSTRALIA

## Abstract

Plants must respond to environmental cues and schedule their development in order to react to periods of abiotic stress and commit fully to growth and reproduction under favorable conditions. This study was initiated to identify SNP markers for characters expressed from the seedling stage to plant maturity in spring and winter wheat (*Triticum aestivum* L.) genotypes adapted to western Canada. Three doubled haploid populations with the winter cultivar ‘Norstar’ as a common parent were developed and genotyped with a 90K Illumina iSelect SNP assay and a 2,998.9 cM consensus map with 17,541 markers constructed. High heritability’s reflected large differences among the parents and relatively low genotype by environment interactions for all characters considered. Significant QTL were detected for the 15 traits examined. However, different QTL for days to heading in controlled environments and the field provided a strong reminder that growth and development are being orchestrated by environmental cues and caution should be exercised when extrapolating conclusions from different experiments. A QTL on chromosome 6A for minimum final leaf number, which determines the rate of phenological development in the seedling stage, was closely linked to QTL for low-temperature tolerance, grain quality, and agronomic characters expressed up to the time of maturity. This suggests phenological development plays a critical role in programming subsequent outcomes for many traits. Transgressive segregation was observed for the lines in each population and QTL with additive effects were identified suggesting that genes for desirable traits could be stacked using Marker Assisted Selection. QTL were identified for characters that could be transferred between the largely isolated western Canadian spring and winter wheat gene pools demonstrating the opportunities offered by Marker Assisted Selection to act as bridges in the identification and transfer of useful genes among related genetic islands while minimizing the drag created by less desirable genes.

## Introduction

Wheat is grown on a larger land area of the world than any other crop [1]. In terms of volume, it is the third largest cereal crop produced after rice and maize and it occupies second place as a food crop for human consumption. Successful wheat production over this wide range of environments has been dependent upon plant breeders’ abilities to find the best combinations of genes with the capacity to respond to regional environmental cues. Most of the wheat is produced in temperate climates where it is subjected to a wide range of stresses. It is normally planted in the spring (spring habit) or in the autumn (winter habit). These differences in growth habit have meant that the winter and spring wheat gene pools have been largely isolated. This isolation has been especially restrictive in regions of the world like western Canada where rigid requirements have been adopted for different grain quality classes of spring and winter wheat. Restraints of this nature on breeding programs support the need to develop efficient methods for the identification and transfer of useful genes among these closely related genetic islands (gene groups that become isolated due to the absence of new genetic variability being introduced into the breeding population) while minimizing the negative consequences created by less desirable genes.

Recent technological innovations allow wheat breeders to improve the efficiency of gene transfer through MAS. In particular, the availability of a chromosomal based draft sequence of the wheat genome [2] and advances in next-generation sequencing have facilitated the discovery of single nucleotide polymorphism (SNP) by whole genome profiling, transcriptome sequencing [3], reduced representation sequencing via genotype by sequencing (GBS; [4]) and exome capture [5];[6]. In most crop species, high-density SNP genotyping arrays are becoming common for studying genomic diversity, inferring ancestral relationships and for identifying marker-trait associations in specialized genetic mapping populations. In wheat, the recent release of the high density SNP iSelect assays (9K- [3] and 90K -[7]) has provided an abundance of markers for detection of quantitative trait loci (QTL) for economically important traits, characterization, and identification of genomic regions targeted by breeding programs and improved selection. Recently, two high density SNP-based consensus maps have been published for hexaploid [7] and tetraploid [8] wheat with >40,000 SNP markers. Association and validation of these markers with commercially important traits should provide a cost effective and efficient means of transferring traits of interest in breeding programs designed to exploit opportunities offered by the wider wheat gene pool.

Genetic and physiological analyses at the whole plant level have shown that rate of phenological development, which is determined by an environmentally regulated genetic system, allows genotypes to position themselves for optimum growth and development in the environment in which they were selected or evolved. In this system, transition from the vegetative to the reproductive growth stage (VRT) is a critical switch that has a major influence on the level of adaptation to different environments (see [9] for a recent review). Length of time in early developmental stages also determines the degree to which the low-temperature (LT) tolerance genetic potential is expressed and plant development toward the VRT in cereals progressively reduces the plant’s ability to cold acclimate [10];[11];[12]. These early physiological and biochemical requirements are therefore critical to the expression of LT tolerance genetic potential. In turn, they have a direct influence on performance during later stages of plant growth and development and, hence, most agronomic and other commercially important traits.

This study was initiated to identify and determine the utility of SNP markers for MAS of a large number of characters expressed from the seedling stage to plant maturity in wheat (Table 1). Simple sequence repeat (SSR), amplified fragment length polymorphism (AFLP), and Diversity Array Technology (DArT) markers were used in initial studies [13];[14] to identify quantitative trait loci (QTL) markers for developmental and cold tolerance related traits in three wheat mapping populations grown in controlled environments. Employing the same mapping populations grown under both controlled environment and field conditions, we have extended those studies using the newly developed 90K wheat SNP array to identify new QTL and confirm the QTL for the traits identified the earlier studies. Field data for winter survival and additional grain quality, yield, and other agronomic traits are also considered with special emphasis on the direct and indirect influence phenological development has on the expression of these characters.

**Table 1:** Phenotypic data for mapping populations.

Trait	winter Manitou	Norstar	Lines Range	Significance	Heritability	Mean	Mid-parent	Standard Error
**Norstar x winter Manitou**								
Controlled Environment Trials								
Final leaf number^w^	16.4	22.1	15.5 to 23.9	>0.0001	0.97	19.6	19.3	0.50
Days to final leaf number^w^	89.8	132.8	89.8 to 146.5	>0.0001	0.96	114.9	111.3	3.60
Days to anthesis^w^	104.9	146.7	103.3 to151.3	>0.0001	0.96	126.8	125.8	4.30
Final leaf number^x^	10.1	13.0	10.0 to 12.7	>0.0001	0.95	11.3	11.6	0.26
Days to final leaf number^x^	21.3	28.7	20.7 to 35.4	>0.0001	0.94	25.1	25.0	0.96
Days to anthesis^x^	33.0	42.0	31.3 to 41.4	0.001	0.94	36.5	37.5	1.15
LT_50_^y^ (°C)	-14.2	-20.7	-12.0 to -22.0	>0.0001	0.98	-16.1	-17.4	0.52
Threshold Temperature	-2.1	-6.0	-2.0 to -6.3	>0.0001	0.93	-3.6	-4.1	0.63
Field Trials								
Field Survival Index (FSI)	401	504	407 to 507	>0.0001	0.98	453	452	5.5
Plant Height (cm)	76	104	78 to 106	>0.0001	0.96	93	90	1.7
Heading Date (doy) ^z^	178.7	181.3	176.4 to 183.0	>0.0001	0.94	179.6	180.0	0.43
Grain Yield (kg/ha)	2350	4576	2811 to 5280	>0.0001	0.96	3578	3463	140.9
1000 Kernel wt (g)	25.9	32.0	23.2 to 35.0	>0.0001	0.98	29.4	29.0	0.40
Protein (%)	16.0	12.3	11.9 to 16.3	>0.0001	0.97	14.2	14.2	0.22
Protein Yield (kg/ha)	381	565	416 to 641	>0.0001	0.90	508	472.7	19.40
**Cappelle Desprez x Norstar**	Cappelle	Norstar	Controlled Environment Trials					
LT_50_ (°C)	-13.1	-20.3	-13.1 to -20.8	>0.0001	0.97	-16.3	-16.7	0.58
**Norstar x Manitou**	Manitou	Norstar	Controlled Environment Trials					
LT_50_ (°C)	-10.0	-20.8	-10.0 to -20.4	>0.0001	0.98	-15.0	-15.4	0.74

^w^ not vernalized

^x^ vernalized

^y^ LT_50_ = Temperature at which 50 percent of the population is killed in an artificial freeze test

^z^ doy = Julian day

## Materials and Methods

### Mapping Populations

The common wheat cultivars Norstar [15], Manitou [16], Cappelle Desprez, and the near-isogenic line (NIL) winter Manitou [17] were used as parents to develop three mapping populations with Norstar as a common parent. These parental lines represent different reproductive strategies and LT tolerance potentials. Norstar is a winter-habit cultivar with a long vernalization requirement that has been a primary source of LT tolerance genes in western Canadian winter wheat breeding programs. Norstar was also the dominant cultivar grown in the Canadian Western Red Winter wheat quality class for many years. Manitou was a widely adapted spring wheat cultivar in the Canadian Western Red Spring (CWRS) wheat quality class. Cappelle Desprez is winter habit European cultivar with a low level of LT tolerance. The difference in vernalization requirement of the winter habit cultivars (*vrn-A1*, *vrn-B1*, *vrn-D1*) and the spring habit Manitou (*Vrn-A1*, *vrn-B1*, *vrn-D1*) is due to a single dominant gene located on chromosome 5A [18]. The reciprocal NIL, winter Manitou, was developed to determine the interactions between LT tolerance and vernalization requirement. Winter Manitou was produced through ten backcrosses of the non-hardy spring habit (*Vrn-A1*) cultivar ‘Manitou’ into the very cold-hardy winter habit (*vrn-A1*) cultivar ‘Norstar’ and selecting for the winter habit v*rn1/vrn1* progeny after each backcross. This procedure resulted a winter habit reciprocal NIL in which theoretically 99.95% of the Manitou parent DNA has been recovered. Three mapping populations were developed using the maize pollination method described by Laurie *et al*. [19]. The Norstar by winter Manitou, Cappelle Desprez by Norstar, and Norstar by Manitou populations consisted of 161, 256, and 152 doubled haploid lines, respectively.

### Controlled Environment Studies

LT_50_ (temperature at which 50% of the plants are killed by LT stress) measurements were determined for the parents and all lines in the three mapping populations using the freeze test procedure described by Limin *et al*. [17]. Experimental design was a three-replicate randomized complete block in which each replicate was separated in time and space. Plants were grown to the three leaf stage under a 20-h day and 4-h night at 20°C and then acclimated at 4°C for 28 days. Crowns of acclimated plants were harvested, covered in moist sand in aluminum weighing cans, and placed in a programmable freezer that was held at -3°C for 12-h. The crowns were cooled at a rate of 2°C h^−1^ down to −17°C and then cooled at a rate of 8°C h^−1^. Five crowns of each line in each replicate were removed from the freezer at 2°C intervals for each of the five pre-selected test temperatures for each line. Samples were then thawed overnight at 4°C, transplanted into trays containing “Redi-earth” (W. R. Grace and Co. of Canada, Ajax, ON, Canada) for regrowth, and placed in a growth room maintained at 20°C with a 20-h day and 4-h night. Plant recovery (alive vs dead) was determined for each line and the parents after three weeks and LT tolerance was expressed as LT_50_.

Winter and spring growth habit was determined for the lines in the Norstar by Manitou population. These lines were grown under a 20-h day and 4-h night at 20°C and those that headed normally compared to Manitou were designated spring habit and those that remained vegetative like Norstar were considered winter habit. Detailed phenological development was only determined for lines in the Norstar by winter Manitou mapping population. Experimental design for these studies was a three-replicate randomized complete block in which each replicate was separated in time and space. Seeds of each line were imbibed in water and held in the dark for 4-d at 4°C, 1-d at 20°C and then planted in 6-inch pots containing Redi-earth. Growth conditions for vernalization were a 20-h day and 4-h night at 4°C for 49-d followed by transfer to 20°C. Lines were also grown under a 20-h day and 4-h night at 20°C to determine development under non-vernalizing conditions. Plant nutrients were supplied using ‘Osmocote’ (Chisso-Asahi Fertilizer Co., Tokyo, Japan) and ‘Tune-up’^TM^, (Plant Products Ltd, Brampton, ON, Canada) as required. Leaves were numbered until the final leaf number (FLN) on the main shoot and days to anthesis could be determined.

Threshold induction temperatures for LT acclimation were determined for lines from the Norstar by winter Manitou mapping population using the plant establishment conditions outlined above for the determination of LT_50_. The experimental design for these studies was a three replicate randomized complete block. The plants were grown under a 16-h day at 20°C until they reached the three leaf stage and they were then transferred to a cold acclimation chamber set at 10°C with 16-h daylight for 2-d. The 10°C acclimation temperature was selected based on the report [20] that the threshold induction temperatures after 2-d of acclimation were 9.3 and 14.7°C for winter Manitou and Norstar, respectively. After the two days of acclimation at 10°C in the present study, LT_50_ was determined for each line in each replicate using the procedure described above. Differences in the LT_50_ values of the lines were then used as estimates of their relative threshold induction temperature, i.e., winter Manitou did not acclimate and lines with a colder LT_50_ were assumed to have started to acclimate earlier based on reported acclimation patterns [20].

### Field Trials

The Norstar by winter Manitou mapping population and its parental cultivars were grown in a total of 17 field trials at Saskatoon (52° N, 107°W; Vertic Haploboroll soil) and Clair (52°N, 104°W; Udic Haploboroll soil), Saskatchewan, Canada, between 2005–06 and 2009–10. The experimental design of all trials was a randomized complete block with two replicates. Analyses of variance were conducted using a mixed effects model where genotypes were considered as fixed and trials as random effects. Plot size was 1.2 m x 6 m with row spacing of 20 cm. Trials were seeded with a no-till small plot hoe-press drill at the optimum seeding date in the autumn [21] and a seeding rate of approximately 215 seeds m^-2^. Phosphate fertilizer (11-51-0 percent N, P_2_O_5,_ and K_2_O, respectively) was applied with the seed and nitrogen (34-0-0) was top-dressed on yield trials as soon as equipment was able to travel on the field in the spring at rates based on soil test recommendations for maximum grain yield. Other elements were not considered limiting. Broadleaf weeds were controlled using recommended post-emergence herbicides applied at the recommended rates and times.

In order to provide a range of over winter stress levels, trials were no-till seeded into standing stubble (NT) from a previous spring sown crop for snow trapping or on summer fallow (SF) fields that were either in the open (O) or protected by shelter belts (SB). Winter kill occurred in twelve trials and provided an effective screen for winter hardiness of the lines in the mapping population (Saskatoon SF 2005–06 (O and SB), Saskatoon NT 2005–06 (O and SB), Saskatoon SF 2006–07, Clair NT 2006–07 (O), Saskatoon SF 2007–08 (O and SB), Saskatoon SF 2008–09 (O and SB), Saskatoon NT 2008–09, Saskatoon SF 2009–10 (O)). After allowing for plant recovery in the spring (approximately three weeks of active growth), winter survival ratings were made using the field survival index (FSI) procedure outlined by Fowler *et al*. [22] to quantify differences in winter hardiness of genotypes. Differences in FSI represent the average percent differences expected in field survival and the higher the FSI, the greater the winter-hardiness potential of a genotype. These trials were abandoned once winter survival ratings were verified by two recorders.

Five trials that did not suffer winter damage (Clair 2006–07 (SB), Clair 2007–08, Saskatoon 2007–08, Clair 2008–09, Clair 2009–10 (NT)) were retained for measurement of date of heading (four sites), grain yield, seed size (1000 kernel weight), grain protein concentration, and grain protein yield (Table 1). Grain yield, kernel weight, protein concentration, and protein yield (grain yield x protein concentration/100) were measured at all five sites. These plots were direct cut at maturity with a self-propelled small plot combine after approximately 30 cm was trimmed from each end. The outside two rows of each plot were not harvested and exact plot lengths were recorded after trimming and before harvest. Grain protein concentration was determined from Leco N x 5.7 (Leco Corp., St. Joseph. MI) (Am. Assoc. Cereal Chem. Method 46–30) for each plot in each trial.

### Genotyping, Linkage and QTL mapping

For all three populations, DNA of parental and DH progeny was extracted from young leaves using the DNeasy 96 Plant Kit (QIAGEN Science, Maryland, USA). DNA concentration was tested using NanoDrop ND-1000 UV–vis spectrophotometer (Thermo Fisher Scientific Inc. Wisconsin, USA). Genotyping was carried out at the Crop Development Centre, University of Saskatchewan using the Illumina Infinium wheat 90K iSelect assay (Illumina Inc., San Diego, CA) as reported previously [7]. The raw intensity data were processed with the GenomeStudio v2011.1 software (Illumina). Genotypic data were curated to remove monomorphic and highly distorted markers according to the expected 1:1 ratio for DH populations, using chi-square (χ^2^) test. For each mapping population, linkage maps were constructed as follows: First, ‘draft’ maps were generated using the MSTMap software [23] with a stringent cut off *p*-value of 1E^-10^ and a maximum distance between markers of 15.0 cM for grouping SNPs into linkage maps. Then, ‘draft’ maps were refined using the MapDisto version 1.7.5 software [24]. A cut off recombination value of 0.35 and threshold LOD score of 3.0 were used. Distances (cM) between markers were calculated using the Kosambi function [25]. Linkage groups (LGs) were checked individually for double recombinants and markers showing double recombination events were re-scored. The best order of markers was generated using both “AutoCheckInversions” and “AutoRipple” commands implemented in the MapDisto version 1.7.5 software [24]. LGs were assigned to chromosomes based on existing high density SNP maps [3];[7];[8].

Because Norstar was a common parent to all three DH populations, a consensus map was built. First, we checked for collinearity and orientation between individual maps using Biomercator [26]. Then, individual maps were integrated into a consensus map using MergeMap [27], a software based on graph theory wherein individual maps are converted into directed acyclic graphs that are then merged into a consensus graph on the basis of their shared vertices [28];[29];[30]. Equal weight (weight = 1.0) was given to all individual maps. MergeMap implements an efficient algorithm for resolving conflicts in the marker order among individual maps by deleting the smallest set of marker occurrences [27]. As MergeMap tends to inflate genetic distances in the consensus genetic map [27];[31], the arithmetic mean of individual maps was used to determine the scaling factor for each linkage group [32]. Marker order was compared between the consensus map and individual maps using Spearman’s rank correlation test.

QTL detection was performed using the inclusive composite interval mapping (ICIM) method as implemented in the Qgene software [33]. The number of marker cofactors for background control was set by forward-backward stepwise regression, with a 10 cM window. The minimum LOD score to declare a putative QTL was obtained after a 1000 permutation test at a 95% confidence level. The QTL Network program version 2.1 [34] based on a mixed-linear model was used to search for epistatic QTL and QTL x environment interactions.

## Results and Discussion

### Phenotypic Assessment

There were significant differences (P<0.001) between the parents of the three mapping populations for all the characters measured in this study (Table 1). These differences were reflected in the wide range of values for the lines in each population. The high level of variability amongst the lines compared to the genotype by trial interaction and error variances resulted in high heritability’s for all characters under consideration. The mapping population mean and mid-parent values were not statistically different (p>0.05) for most characters suggesting that their expression was mainly determined by additive gene action. There was transgressive segregation for many of the characters.

#### a) Phenological Development

Phenological development is determined by a complex, environmentally regulated genetic system. The early stages of phenological development are primarily a function of (i) minimum final leaf number (MFLN), (ii) length of plastochron, (iii) vernalization (cold) requirements, (iv) photoperiod (day length) restraints, and (v) temperatures near freezing that delay the vegetative to the reproductive transition (VRT) in plants that have reached the stage of competence to flower [9]. These requirements must all be met before the plant will enter the reproductive stage. Plant development and time to the VRT growth stage in fully vernalized genotypes grown under long days is dependent on highly heritable, temperature-dependent genotypic differences that determine the MFLN and length of the plastochron (the interval between successive leaf appearances). The length of the plastochron is dependent on both time and temperature [35];[36] and is normally measured by accumulated thermal units, such as growing degree days. Plants with a vernalization requirement need a period of exposure to cold temperatures in the autumn for normal growth and development under field conditions. A photoperiod response in cereals normally serves to delay the VRT until the lengthening days of spring signal the start of the summer growing period. Vernalization and photoperiod requirements both act to lengthen the vegetative growth phase, primarily through mechanisms that increase the number of leaves produced on the main stem before transition to the reproductive stage.

The vernalization requirement of both Norstar, winter Manitou, and their progeny was reflected in the reduced final leaf number, days to final leaf number, and days to anthesis for plants vernalized at 4°C compared to those grown continuously at 20°C (Table 1). Significant correlation coefficients (P<0.01) indicated an underlying relationship among these characters when plants were grown in the controlled environment conditions utilized in this study (Table 2). This suggests that at least part of their expression has a common genetic basis.

**Table 2:** Phenotypic correlations among a) final leaf number (FLN), days to final leaf number (DFLN), and days to anthesis (DA) for plants that were not vernalized, b) final leaf number (FLNv), days to final leaf number (DFLNv), and days to anthesis (DAv) for plants that were vernalized, and c) LT_50_, threshold induction temperature (Thres), field survival index (FSI), plant height (HT), heading date (HD), maturity (MAT), grain yield (YIELD), 1000 Kernel wt (1000K), protein content (PROT%), Protein Yield (PTYD) for the Norstar by winter Manitou mapping population.

	FLN	DFLN	DA	FLNv	DFLNv	DAv	LT_50_	Thres	FSI	HT	HD	YIELD	1000K	PROT%
DFLN	**0.83**^z^													
DA	**0.76**	**0.89**												
FLNv	**0.62**	**0.47**	**0.54**											
DFLNv	**0.40**	**0.45**	**0.49**	**0.51**										
DAv	**0.29**	**0.35**	**0.40**	**0.36**	**0.89**									
LT_50_	**-0.30**	*-0*.*27*	**-0.28**	**-0.32**	0.16	-0.10								
Thres	**-0.30**	**-0.33**	**-0.34**	**-0.38**	*-0*.*22*	-0.13	**0.55**							
FSI	*0*.*27*	0.16	0.13	**0.28**	0.11	0.11	**-0.46**	**-0.27**						
HT	**0.45**	**0.34**	**0.43**	**0.29**	*0*.*25*	0.20	-0.16	-0.18	0.03					
HD	0.12	*0*.*22*	*0*.*27*	0.10	**0.39**	**0.49**	0.18	0.08	**-0.28**	*0*.*25*				
YIELD	**0.37**	**0.41**	**0.43**	**0.45**	*0*.*26*	0.16	*-0*.*25*	**-0.38**	0.12	*0*.*24*	*0*.*24*			
1000K	-0.12	-0.10	-0.02	0.07	-0.03	-0.11	-0.07	-0.05	-0.08	0.03	-0.13	0.20		
PROT%	**-0.32**	**-0.41**	**-0.46**	**-0.36**	**-0.32**	**-0.29**	0.19	**0.34**	0.01	**-0.34**	**-0.47**	**-0.68**	0.07	
PYLD	**0.29**	**0.29**	**0.30**	**0.35**	0.16	0.04	-0.20	**-0.28**	0.14	0.12	0.03	**0.89**	**0.29**	**-0.28**

^**z**^ Significance of correlation coefficient

Bold = 0.001, Italics = 0.01, Shaded = 0.05

Heading date is influenced by many genes, the most significant of which are vernalization, photoperiod, and earliness (for a recent review see [37]). In the present study, plants in controlled environments were grown in uniform conditions under a 20-h day length while those in the field experienced shorter days and a much more variable temperature range. Norstar was later than winter Manitou in all environments (Table 1) but, while significant, relatively small correlation coefficients (Table 2) for heading date under field conditions and days to anthesis of vernalized and nonvernalized plants in controlled conditions indicated that plant development did not progress the same in these different environments.

#### b) Low-Temperature Tolerance

A complex, environmentally driven genetic system, which is developmentally regulated and induced by exposure to LT [10];[11], is responsible for cold acclimation in cereals. As outlined earlier, development toward flowering progressively reduces the plant’s ability to LT acclimate and the VRT is a critical switch that initiates the down regulation of LT tolerance genes [10];[11];[12]. As a result, it has been difficult to separate cause and effect adjustments to LT and other environmental cues that signal seasonal changes [38].

Norstar is a winter habit cultivar while Manitou is a spring wheat. Manitou is known to carry a single dominant gene, *Vrn-A1*, which determines spring habit [18]. Low temperature acclimation and vernalization are induced by temperatures that fall in a similar range, and the LT requirement for cold hardening is also the cue that results in the conversion of winter cereals to the spring form. In the Norstar by Manitou mapping population, 65 of the doubled haploid lines were spring and 87 were winter habit. The LT_50_ range was -20.3 to -11.4 for the winter and -14.0 to -9.3°C for the spring habit lines. While there was an overlap phenotypic expression, these differences reflected the longer vernalization requirement and the expected associated LT tolerance gene up-regulation for the winter habit lines [11]. The mid-parent and mean LT_50_s of the populations were -15.3 and -15.5°C for the winter lines and -11.3 and -11.3°C for the spring habit lines. This indicates that, while the differences in LT_50_ due to vernalization requirement were determined by a single dominant gene, the remaining variability for this character was primarily controlled by additive gene action. Observations made on the Cappelle Desprez by Norstar mapping population support this observation (Table 1). However, it must be noted that the mid-parent LT_50_ was 1.3°C colder than the mean value in the Norstar by winter Manitou mapping population, where the effect of the vernalization gene was neutralized, suggesting that additional non-additive affects may also play a role. The Norstar and winter Manitou mapping population was grown under long days (20-h) in the controlled environment studies. Consequently, the main difference in their rate of phenological development was determined by a larger MFLN for Norstar (Table 1). A larger MFLN and the associated delay in development would be expected to extend the period of LT tolerance gene up-regulation [17], but it is unlikely that this difference alone would explain the cold tolerance advantage of Norstar compared to winter Manitou.

The superior cold tolerance of Norstar was reflected in a colder LT_50_ than Manitou, Cappelle Desprez and winter Manitou (Table 1). A close relationship has been reported between the minimum LT_50_ that a genotype can achieve and both winter survival under field conditions in western Canada [39] and the threshold temperature at which cold acclimation starts [20]. Highly significant correlations coefficients were observed among these characters for the lines in the Norstar by winter Manitou mapping population (Table 2), but the unexplained variability was large enough to suggest that LT_50_ was not the only factor determining winter survival potential of a wheat genotype.

#### c) Plant Height, Grain Yield, Kernel Weight, and Grain Protein

There are three primary yield component traits in wheat at normal planting densities; tillers per plant, seeds per spike, and 1000 kernel weight (seed size). The number of fertile tillers per plant is normally determined early in the growing season, followed by the number of seeds per spike, and then finally seed size (see [40] for a review related to western Canadian environments). The developing crop makes adjustments to adverse environmental conditions encountered during the growing season by retarding formation of the most actively developing yield component at the time of stress [41];[42]. A large number of genes with complex genotype by environment interactions usually results in low heritability’s for grain yield. Although large differences are normally expected in weather conditions for different locations and years in western Canada, the environments we sampled were relatively stable and the genotype by location year interaction was insignificant. The resulting high heritability’s indicate that the variability observed for grain yield and related characters was mainly due to genetic differences in the present study (Table 1).

Neither parent in the Norstar by winter Manitou mapping populations carry the major reduced height (*Rht*) dwarfing genes [43];[44], however; Norstar was 28 cm taller than winter Manitou (Table 1). The height difference of the parents was reflected in the range observed in the progeny while similar mean and mid-point heights suggested that this character was primarily under additive genetic control. The grain yield and kernel weight of Norstar were considerably higher than that of winter Manitou. The range of kernel weight for the lines in this mapping population fell outside the values for the parents while all the lines had higher yield than winter Manitou. A few lines had a heavier seed weight and were higher yielding than Norstar. There was a small, but significant correlation between seed weight and grain yield (Table 2) for the lines in Norstar by winter Manitou mapping population.

High protein content is a quality requirement of CWRS wheat quality class and this standard was reflected in the much higher value for winter Manitou compared to Norstar (Table 1). Protein yield is a direct function of available soil nitrogen, which can be considered a constant for genotypes growing in the same field trial. This results in a negative correlation between grain yield potential and protein content (Table 2) and any increase in cultivar grain yield potential is expected to result in lower protein content unless a cultivar has an increased ability to utilize available soil N for protein production (protein content (%) = grain protein yield /grain yield x 100). In this regard, winter Manitou had a much higher protein concentration than Norstar, but a lower grain protein yield for winter Manitou indicated that Norstar had a superior ability to utilize available soil N for protein production.

### High density consensus map

Prior to the consensus map, individual maps were built for each population. S1, S2 and S3 Tables give the genotypic data and the maps of Norstar by Cappelle Desprez, Norstar by Manitou and Norstar by winter Manitou, respectively. For Norstar by Cappelle Desprez population, the map consisted of 10,253 markers that span 3,147.2 cM. The map of Norstar by Manitou consisted of 7,971 markers spanning 2,946.2 cM. A total of 9,487 markers were mapped in Norstar by winter Manitou with a map length of 2,829.0 cM. Despite the varying number of markers and map length between populations, the average markers density (0.3) was similar among populations.

The three individual maps were successfully merged into a consensus map for the hexaploid wheat genome using the graph theory [28];[29];[30] as implemented in MergeMap [27]. This was achieved due to the presence of many (11,829) anchor points between populations; there were 2467, 2630, and 6732 anchor points between Norstar by Cappelle Desprez and Norstar by Manitou, Norstar by Cappelle Desprez and Norstar by winter Manitou, and Norstar by Manitou and Norstar by winter Manitou, respectively. The graph theory algorithm was used for generating high density SNP-based consensus map for crops such as barley [31];[32] and cowpea [45]. The consensus map consisted of 17,541 markers spanning all common wheat chromosomes except 6D (Table 3). The total length of the map was 2,998.9 cM with an average density of one marker per 0.2 cM. The map was then used to investigate characters expressed from the seedling stage to plant maturity in our three mapping populations. Most markers were relatively evenly distributed along the chromosomes, although some regions showed gaps. The biggest gap (27.1 cM) was located on chromosome 1A. The order of markers on the consensus map was in good agreement (Spearman’s rank correlation ranging from 0.88 to 0.99, p-value < 0.0001) with the corresponding order of the individual linkage maps.

**Table 3:** Features of the consensus map.

Chromosome	Number of markers	Length(cM)	Density
1A	1228	149.4	0.1
2A	771	194.5	0.3
3A	872	201.7	0.2
4A	764	185.4	0.2
5A	1212	249	0.2
6A	1374	134	0.1
7A	1026	204.7	0.2
**Total Genome A**	**7247**	**1318.7**	**0.2**
1B	1114	212.8	0.2
2B	2273	150.9	0.1
3B	1173	206.4	0.2
4B	630	156.8	0.2
5B	1521	186.2	0.1
6B	1415	188.3	0.1
7B	1000	149.8	0.1
**Total Genome B**	**9126**	**1251.2**	**0.1**
1D	232	84.9	0.4
2D	597	132.1	0.2
3D	122	39.7	0.3
4D	47	62.3	1.3
5D	66	30.9	0.5
7D	104	79.1	0.8
**Total Genome D**	**1168**	**429**	**0.4**
Total	17541	2998.9	0.2

Individual genetic maps have been published for hexaploid wheat with genetic distances ranging from 2,060 cM [46] to 5,332 cM [47]. More recently, a genetic map of 3,930 cM has been published [48]. To our knowledge, only few consensus maps have been built for hexaploid wheat [7];[49];[50]. Our consensus map is 21% shorter than that of Wang *et al*. [7] (2,998.9 versus 3,800.6 cM) although those authors had corrected the final map length for inflation rates. The relatively short length of our map could be due to the fact that only few (1,168) markers were mapped on the D genome, spanning 429 cM. It is well known that the D-genome of wheat varieties harbors limited marker polymorphism [51]. This phenomenon appeared to be exacerbated in our material for chromosome 6D for which we could not build a composite map. The chromosome 6D was reported to have the lowest number of markers and to be the shortest of the high-density maps of the wheat genome [51]. Despite these differences in marker number and map size, there was similar marker density between our consensus map and that of Wang *et al*. [7]. In total, 18% (3,208) of markers from our map were not present in their map, despite its higher (> 40,000) number of markers. The order of markers was highly correlated (Spearman’s rank correlation = 0.87, p-value < 0.0001) between the two maps.

### QTL

#### a) Phenological Development

**Fig 1:**
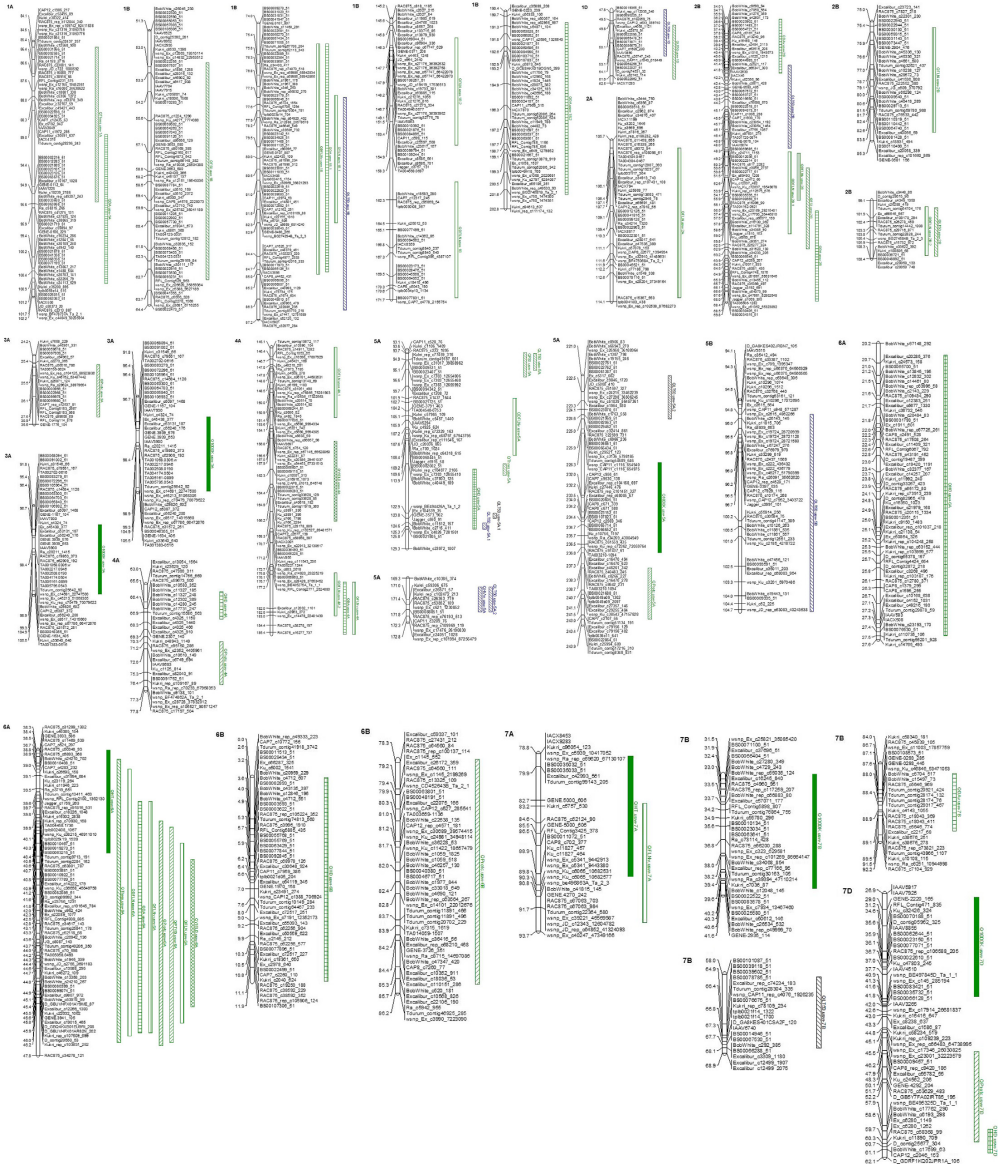
QTL for phenological development, low-temperature tolerance, quality, and yield-related traits across three mapping populations [Norstar x winter Manitou (green), Norstar x Manitou (Blue), and Cappelle Desprez x Norstar (black)]. a) Final leaf number (FLN), days to final leaf number (DFLN), and days to anthesis (DA) for plants that were not vernalized, b) final leaf number (FLNv), days to final leaf number (DFLNv), and days to anthesis (DAv) for plants that were vernalized, and c) LT_50_, threshold induction temperature (Thres), field survival index (FSI), habit *Vrn*-A1 (HVrn), plant height (HT), heading date (HD), maturity (MAT), grain yield (YIELD), 1000 Kernel wt (1000K), protein content (Protc), Protein Yield (PTYD).

There were a number of significant QTL detected for each measure of phenological development considered (Table 4, Fig 1) in the controlled environment studies. QTL on chromosomes 1B, 4A, and 6A were frequently detected for measures related to final leaf number and days to anthesis. However, there were also a large number of additional QTL that were more specific in their association with these characters.

**Table 4:** QTL Norstar by winter Manitou summary. Position (cM) is given at the peak of LOD with the confidence interval (cM). Additive effect is given according to the parent, Norstar (a) and winter Manitou (b).

Trait	QTL	Chromosome	Position (confidence interval)	Additive effect	LOD	R^2^
**Controlled Environment Trials**						
1. Final leaf number nonvernalized	QFLN.usw-1B.1	1B	80.0 (5.7)	0.515^a^	6.659	20.1
	QFLN.usw-1B.2	1B	152.0 (10.1)	0.401^b^	3.617	11.4
	QFLN.usw-2A	2A	110.0 (7.8)	0.435^b^	4.747	14.7
	QFLN.usw-2B	2B	80.0 (9.1)	0.943^b^	4.007	12.6
	QFLN.usw-6A	6A	44.0 (5.6)	0.747^a^	6.783	20.4
2. Days to FLN nonvernalized	QDFLN.usw-1B	1B	166.0 (10.9)	2.75^b^	3.175	10.5
	QDFLN.usw-2B.1	2B	56.0 (10.4)	3.276^b^	3.352	11.0
	QDFLN.usw-2B.2	2B	104.0 (7.8)	4.093^a^	4.541	14.7
	QDFLN.usw-4A.1	4A	150.0 (10.0)	3.637^b^	3.490	11.5
	QDFLN.usw-4A.2	4A	180.0 (6.1)	4.95^a^	6.020	18.9
	QDFLN.usw-6A	6A	44.0 (10.3)	3.849^a^	3.390	11.2
3. Days to anthesis nonvernalized	QDA.usw-1A	1A	96.0 (10.6)	8.832^b^	3.475	10.8
	QDA.usw-1B.1	1B	80.0 (10.2)	8.651^a^	3.661	11.3
	QDA.usw-1B.2	1B	196.0 (11.8)	2.375^b^	3.115	9.7.0
	QDA.usw-2B	2B	64.0 (11.4)	2.832^b^	3.201	10.0
	QDA.usw-4A	4A	182.0 (7.4)	3.004^a^	5.156	15.6
	QDA.usw-6A	6A	44.0 (8.2)	4.113^a^	4.568	14.0
4. Final leaf number vernalized	QFLNv.usw-4A	4A	182.0 (7.4)	0.127^a^	2.811	8.9
	QFLNv.usw-6A	6A	44.0 (8.2)	0.266^a^	6.404	19.1
	QFLNv.usw-7A	7A	88.0 (10.0)	0.151^a^	3.688	11.5
5. Days to FLN vernalized	QDFLNv.usw-1B	1B	80.0 (10.3)	0.523^a^	2.949	9.3
	QDFLNv.usw-2B	2B	54.0 (6.0)	0.799^a^	6.420	19.2
	QDFLNv.usw-5A	5A	107.2 (10.4)	0.598^a^	3.501	11.0
6. Days to anthesis vernalized	QDAv.usw-2B	2B	36.0 (9.8)	0.688^a^	4.000	12.3
	QDAv.usw-7B	7B	88.0 (6.1)	0.629^a^	4.000	12.3
7. LT_50_ (°C)	QLT50.usw-1D	1D	56.0 (11.3)	0.775^b^	3.263	10.2
	QLT50.usw-2B	2B	106.0 (11.4)	1.135^a^	3.200	10.1
	QLT50.usw-5A	5A	95.0 (2.4)	1.424^b^	20.110	48.6
8. Threshold Temp (°C)	QThres.usw-1A	1A	90.0 (11.0)	0.323^a^	3.374	10.4
	QThres.usw-5A	5A	113.9 (2.7)	0.666^b^	16.786	42.0
	QThres.usw-6A	6A	44.0 (10.8)	0.349^b^	3.441	10.6
**Field Trials**						
9. Field survival index	QFSI.usw-1B	1B	58.0 (10.4)	5.667^a^	3.576	11.0
	QFSI.usw-2B	2B	58.0 (10.2)	16.839^a^	3.702	11.3
	QFSI.usw-5A	5A	95.0 (6.8)	8.134^a^	5.672	16.8
10. Height (cm)	QHT.usw-6A	6A	38.0 (3.5)	2.968^a^	12.080	32.4
	QHT.usw-7A	7A	84.0 (10.1)	1.347^a^	3.737	11.4
11. Heading Date (doy)	QHD.usw-4A	4A	66.0 (3.8)	0.764^a^	10.990	30,0
	QHD.usw-5A	5A	123.0 (10.1)	0.325^b^	3.727	11.4
	QHD.usw-6A	6A	22.0 (11.0)	1.500^a^	3.384	10.4
	QHD.usw-6B	6B	6.0 (13.5)	0.267^a^	2.756	8.5
	QHD.usw-7D	7D	60.0 (3.2)	0.648^a^	13.942	36.4
12. Grain Yield (kg/ha)	QYIELD.usw-4A	4A	164.0 (11.4)	199.951^a^	3.272	10.1
	QYIELD.usw-6A	6A	44.0 (4.6)	196.26^a^	8.811	24.9
13. 1000 Kernel wt. (g)	Q1000K.usw-3A	3A	100.0 (10.7)	0.675^b^	3.49	10.7
	Q1000K.usw-5A	5A	233.5 (8.3)	0.619^a^	4.616	13.9
	Q1000K.usw-7B	7B	36.0 (9.9)	0.568^a^	3.793	11.6
	Q1000K.usw-7D	7D	34.0 (13.2)	0.467^b^	2.793	8.7
14. Protein Content (%)	QProtc.usw-3A	3A	30.0 (8.6)	0.172^a^	4.421	13.4
	QProtc.usw-4A	4A	74.0 (6.0)	0.376^b^	6.516	19.0
	QProtc.usw-5A	5A	240.7 (4.7)	0.249^a^	8.534	24.2
	QProtc.usw-6A	6A	44.0 (7.0)	0.303^b^	5.501	16.3
	QProtc.usw-6B	6B	82.0 (7.7)	0.206^b^	5.025	15.0
	QProtc.usw-7D	7D	54.0 (12.1)	0.138^b^	3.079	9.5
15. Protein Yield (kg/ha)	QPTYD.usw-6A	6A	44.0 (6.7)	17.871^a^	5.819	17.2

^a^ Additive effect of Norstar

^b^ Additive effect of winter Manitou

There were five QTL for final leaf number of nonvernalized plants mapped to four chromosomes in the present study. Using the same mapping population in a study that employed 678 microsatellite and 488 Diversity Array Technology (DArT) markers, Baga *et al*. [13] reported QTL for this character on chromosomes 1B, 2A, 2B, 5A, 6A, and 7A. This brought the total number of QTL for this character to eight for both studies. Only three QTL were identified for final leaf number of vernalized plants in the current study. Baga *et al*. [13] had also identified QTL on the same chromosomes and reported a total of seven QTL for this character, all on different chromosomes.

A total of six QTL on four chromosomes were identified for days to FLN of nonvernalized plants in the current study. These were located on chromosomes 1B, 2B (two), 4A (two) and 6A. Seven QTL located on 1B (two), 2B (two), 4A, 6A, and 6D were reported by Baga *et al*. [13] for this character. Eight QTL were reported on six chromosomes for days to FLN of vernalized plants. Both studies had QTL on chromosomes 1B and 2B. Baga *et al*. [13] reported QTL on 4A, 6B, and 6D and there was a 5A QTL detected in the present study.

The major vernalization genes have been located to chromosomes 5A (*Vrn-A1* and *Vrn-A2*) and 7BS (*Vrn-A3*). The primary photoperiod genes map to 2A (*Ppd-A1*), 2B (*Ppd-B1*), 2D (*Ppd-D1*), 7A (*Ppd-B2*), and 7BS (*Ppd-B3*) while the earliness genes are found on chromosomes 1A and 3A. McCartney *et al*. [52] also reported maturity QTL on chromosomes 7D, 4A, 4D, and 3B in a mapping study that used spring wheat parents which represented two western Canadian marketing classes. In an association mapping study using the 90K iSelect wheat chip, Zanke *et al*. [37] identified developmental associations on all chromosomes in European winter wheat. The highest number of marker-trait associations was on chromosome 5B. The photoperiod (*Ppd-A1*, *Ppd-B1*, and *Ppd-D1*) and vernalization (*Vrn-A2*) genes were found to have a major influence and they concluded that many more genes were involved in determining heading date.

QTL reported on chromosomes 2B, 4A, and 6A for days to anthesis of nonvernalized plants by Baga *et al*. [13] were also detected in the present study. Three additional QTL for this character were observed on two different chromosomes in the present study. Seven QTL on six chromosomes were noted for days to anthesis of vernalized plants. 2B was the only chromosome with a QTL reported in both studies.

Six QTL were located to five different chromosomes (1A, 1B (two), 2B, 4A, and 6A) for days to anthesis in nonvernalized plants grown in controlled environments (Table 4) in the present study. In contrast, QTL were only found on chromosomes 2B and 7B for days to anthesis when plants grown in controlled environments were vernalized. There were no QTL on the same chromosomes for days to anthesis of vernalized plants grown in controlled environments compared to heading date under field conditions (4A, 5A, 6A, 6B, and 7D). This suggests that there were major differences in the way phenological development progressed in these two very different environments.

#### b) Low-Temperature Tolerance

SNP markers were associated with a total of eight different QTL for LT_50_ (Table 4, Fig 1). The QTL on chromosome 5A at position 123/125 in the Cappelle Desprez by Norstar (Table 5) and Norstar by Manitou (Table 6) and at position 95 in the Norstar by winter Manitou (Table 4) population in the region of the *Fr2* loci [14];[53] were the only QTL common to the three populations considered in the present study. A single major QTL for the vernalization growth habit gene, v*rn-A1*, located on chromosome 5A was also the position of a major QTL for LT_50_ identified in the Norstar by Manitou mapping population (Table 6). The 5A *Fr2* associated QTL in all three mapping populations and the QTL in the v*rn-A1* region were the major factors determining LT_50_ in these studies.

**Table 5:** QTL of Cappelle Desprez x Norstar detected in controlled Environment Trials. Position (cM) is given at the peak of LOD with the confidence interval (cM). Additive effect is given according to the parent, Norstar (a) and Cappelle Desprez (b).

Trait	QTL	Chromosome	Position (confidence interval)	Additive effect	LOD	R^2^
LT_50_ (°C)	QLT50.usw-3A	3A	160.0 (13.0)	0.239^a^	2.790	5.2
	QLT50.usw-5A.1nc	5A	123.0 (1.3)	1.229^b^	38.671	52.2
	QLT50.usw-5A.2nc	5A	222.5 (3.7)	0.91^b^	10.423	18.1
	QLT50.usw-7B	7B	63.3 (9.8)	0.285^b^	3.731	6.9

^a^ Additive effect of Norstar

^b^ Additive effect of Cappelle Desprez

**Table 6:** QTL of Norstar x Manitou detected in controlled Environment Trials. Position (cM) is given at the peak of LOD with the confidence interval (cM). Additive effect is given according to the parent, Norstar (a) and Manitou (b).

Trait	QTL	Chromosome	Position (Confidence interval)	Additive effect	LOD	R^2^
LT_50_	QLT50.usw-1B	1B	82.0 (9.5)	0.261^b^	3.937	11.7
	QLT50.usw-1D	1D	52.0 (9.7)	0.239^b^	3.856	11.5
	QLT50.usw-2B	2B	45.1 (10.5)	1.390^b^	3.548	10.6
	QLT50.usw-5A.1nm	5A	125.0 (1.4)	1.168^b^	44.794	75.7
	QLT50.usw-5A.2nm	5A	171.0 (1.4)	1.790^b^	49.222	78.8
	QLT50.usw-5B	5B	99.8 (10.0)	0.289^b^	3.773	11.2
Habit *Vrn-*A1	QHVrn-5A	5A	78.0 (1.4)	0.472^a^	70.976	89.3

^a^ Additive effect from Norstar

^b^ Additive effect from Manitou

It should be noted the Norstar x Manitou population is essentially the same as the Norstar by winter Manitou population, but segregates for the *VRN-A1* spring versus winter growth habit allele. However, different QTL for low-temperature tolerance were identified in the Norstar x Manitou population (1B, 1D, 2B, 5A, 5A, 5B) versus the Norstar by winter Manitou population (1D, 2B, 5A). While winter Manitou has an apparent single gene substitution, it should not come as a surprise that including the *VRN-A1* allele for spring growth habit would lead to larger changes in LT tolerance gene expression. *VRN-A1* influences the VRT and Laudencia-Chingcuanco *et al*. [54] have reported that substitution of the spring with the winter habit gene in Manitou resulted in the differential expression of 4,064 genes (p < 0.01) on the Affymetrix microarray during cold acclimation/vernalization indicating a large number of metabolic pathways and cellular processes are influenced by the vernalization requirement.

Baga *et al*. [14] reported QTL for LT_50_ on chromosomes 1D (two), 2A, 2B, 5A, 6D, and 7B in the Norstar by winter Manitou population giving a total of 11 QTL for LT_50_ in all studies using these mapping populations. QTL on 1D and 2B were observed in all cases except for the Cappelle Desprez by Norstar population, while QTL on chromosomes 1D, 2B, and 5A were reported for the Norstar by winter Manitou population in both marker studies. Among all the characters considered, the only epistatic QTL interaction was an additive x additive interaction between QTL for LT_50_ on chromosomes 1B and 2B in the Norstar by Manitou mapping population (Table 7). However, while this interaction was significant, its heritability was very low suggesting it was of relatively minor importance in MAS programs.

**Table 7:** Epistatic QTL interactions for LT_50_ in Norstar x Manitou detected in controlled Environment Trials. Additive x additive interaction effects (AA) and their heritability h^2^ (aa) are shown.

Population	Trait	QTL_i	QTL_j	AA	P-value	h^2^(aa)
		Chromosome (Position)	Position (cM)			
Norstar x Manitou	LT_50_	2B (45.1)	2B (107.1)	-0.24	0.003	0.01
		1B (82.0)	2B (107.1)	-0.22	0.026	0.01

A close relationship has been reported between the threshold temperature at which acclimation starts and the minimum LT_50_ that a genotype can achieve (Table 2; [20]). However, the only major QTL observed in a similar position for both minimum LT_50_ and threshold temperature was on chromosome 5A (Table 4) suggesting that additional different mechanisms also play a role in the expression of these two characters.

LT tolerance is not the only factor determining winter survival of a wheat genotype. However, LT stress is the primary over winter stress in western Canada and a close relationship between LT_50_ and field survival has been demonstrated ([39]; Table 2). The major QTL on chromosome 5A and a smaller effect QTL on chromosome 2B for both characters are the probable reasons for this close association (Table 4). An additional QTL for field survival on chromosome 1B suggests that there are also different factors determining part of the variation in these two characters.

#### c) Plant Height, Grain Yield, Kernel Weight, and Grain Protein

Major genes for reduced height (semi-dwarf) are located on chromosomes 1B (*Rht-B1*), 1D (*Rht-D1*), and 2D (*Rht8*) [55] in hexaploid wheat. A large number of smaller effect QTL that represent nearly every chromosome in wheat have also been reported [52];[56];[57]. None of the parents utilized in developing the Norstar by winter Manitou mapping populations in the present study were semi-dwarf. However, there were height differences between the parents (Table 1) and a major height QTL was found on chromosome 6A and a second QTL with a lesser effect was located on chromosome 7A (Table 4).

Even though grain yield is one of the most thoroughly studied characters, it is still poorly understood. Zhang *et al*. [57] surveyed the published reports relating to grain yield and yield component QTL. A large number of QTL that were unevenly distributed on all 21 wheat chromosomes had been reported in the literature. Important genomic regions for yield related characters were found on chromosomes 1BL, 2AS, 2DS, 3B, 4A, 4B, 4D, and 5A. These QTL were often reported in association with major growth and development genes, such as vernalization, photoperiod [58] and plant height [52], that are known to influence grain yield. Important QTL for grain yield have also been reported on chromosomes 6A, 6B, and 6D [59]. Grain yield QTL were only observed on chromosomes 4A and 6A in the present study (Table 4).

The literature review by Zhang *et al*. [57] indicated that there were a large number of kernel weight QTL, with important ones on chromosomes 1B, 3B, and 4AL. Additional QTL on chromosomes 6B and 7A have been reported for kernel mass by Raman *et al*. [60] in an Australian Chara/WW2449 mapping study. QTL for kernel weight were observed on chromosomes 2A, 3D, 4A, 4B, 4D, and 6D in a spring wheat mapping study with parents from two western Canadian marketing classes [52]. Manitou, which was the recurrent parent in the development of winter Manitou used in the present study, and ‘AC Domain’, one of the parents used in the McCartney study, both belong to the CWRS wheat quality class that has kernel weight requirements. However, none of the kernel weight QTL (Table 4) reported in these two studies were located on the same chromosomes.

A major grain protein content and protein yield QTL at the same location on chromosome 6A (Table 4) indicates that the lower protein content of Norstar was primarily due to a grain yield dilution effect. Although not detected for grain protein yield, a second minor QTL for total grain yield was also located on chromosome 4A. A QTL for grain protein content has also been reported on chromosome 4A by Raman *et al*. [60]. QLT located on chromosomes 3A, 4A (different position from the yield 4A in the present study), 5A, 6B, and 7D offer the possibility to increase the ability to utilize available soil N for protein production thereby increasing protein content without a corresponding decrease in grain yield. A genomic region on chromosome 3A plus a second QTL on 5D have also been reported for protein content in earlier studies [58].

## Summary

This study considered a large number of characters that were expressed from the seedling stage to plant maturity (Table 1). Significant correlation coefficients for many characters, including some that were measured at much different times in the life cycle (Table 2), suggested that at least part of their expression had a common genetic basis. High heritability’s for all characters were a reflection of the large differences between the parents and relatively low genotype by environment interactions for plants grown in controlled environments or in the field. However, the absence of QTL in common for days to anthesis of vernalized plants grown in controlled environments compared to heading date under field conditions reminds us that the speed and direction of growth and development are being orchestrated by environmental cues and we should be cautious when extrapolating conclusions from controlled environment studies to the field. These observations are similar to reports that different genes determine flowering time under different environments in *Brassica* species [60];[61].

As expected, there were differences in QTL and in map locations among the mapping populations and marker types considered. A significant QTL on chromosomes 6A influenced the expression of a number of characters while two QTL on chromosome 5A had a major influence on phenological development and LT tolerance in the present study (Table 4). The major 5A QTL were associated with the vernalization gene, v*rn-A1*, and the LT tolerance locus, *Fr2*. Minor effect QTL were detected for all characters in the mapping populations, but there was considerable unexplained heritability remaining for most characters. The mean and mid- parent values were similar for most characters indicating that their expression was mainly determined by additive gene action. This suggests that the genes and alleles responsible for these characters could be stacked using MAS.

The QTL for vernalization on chromosome 5A (v*rn-A1*) was associated with both phenological development and LT_50_ in the Norstar by Manitou mapping population (Table 6). This close relationship has been attributed to tight linkage of v*rn-A1* and a cold tolerance gene designated *Fr1* [62] or a pleiotropic effect of v*rn-A1*[18]. The pleiotropic effect of v*rn-A1* has been shown to act through its influence on the transition of the plant from the vegetative to the reproductive stage that also down regulates the expression of the LT tolerance genes [10];[11];[12]. It should be noted that a LT tolerance QTL was not observed for the *Fr1* position in the Norstar by winter Manitou mapping population, even though the winter Manitou parent was developed using 10 backcrosses to Manitou, suggesting the absence of a gene for LT tolerance linked to v*rn-A1* or the presence of the same allele in both parents. A major QTL for LT_50_, threshold induction temperature, and field survival index near the *Fr2* position of chromosome 5A was common to all mapping populations. A number of smaller effect QTL were detected, but these were specific to the different mapping populations. Among all the characters considered in this study, the only epistatic interaction detected was an additive x additive interaction between QTL for LT_50_ on chromosomes 1B and 2B in the Norstar by Manitou mapping population (Table 7).

The QTL on chromosome 6A in the Norstar x winter Manitou population was particularly interesting in that it was associated with days to anthesis, days to final leaf number (FLN), and FLN under non-vernalizing conditions and FLN under conditions for vernalization in a controlled environment (Table 4). MFLN determines the shortest time required for a plant to reach the VRT growth stage [9]. Norstar has a higher MFLN on the main stem than Manitou (Table 1), which is associated with a delay in the phenological development of Norstar. Because there is a down regulation of the LT tolerance gene expression associated with the VRT, plants with a similar LT tolerance genetic potential and a higher MFLN have a longer time to express this potential. This QTL was also associated with the threshold induction temperature, grain yield, grain protein content, grain protein yield, and perhaps plant height. More leaf nodes on the stem is expected to produce taller plants unless the distance between the internodes changes. The relationship with the remaining characters was not as clear, but is it possible that they are also being influenced by a delay in the VRT and the associated increased opportunity to accumulate biomass before a change in emphasis from vegetative to reproductive priorities. In this scenario, transfer of the early season delay in phenological development from winter wheat may be of limited value for characters associated with grain yield improvement in western Canadian spring wheat breeding programs if it was accompanied by a delay in maturity and a protein concentration dilution effect. However, it must be noted that the heading of Norstar was less than three days later than winter Manitou (Table 1) suggesting that a slower rate of phenological develop early in the growing season, when temperatures are cooler, may not necessarily translate into a significant delay in maturity. Also, QTL located on chromosomes 3A, 4A (different position from the yield 4A), 5A, 6B, and 7D offer the possibility to increase protein content without a corresponding decrease in grain yield.

A large number of SNP QTL markers have been identified for genes of interest that could be transferred between the largely isolated western Canadian spring and winter wheat gene pools using MAS methods. There was transgressive segregation for the lines in each mapping population and QTL with additive effects were identified for all of the characters considered. This ever increasing number of QTL for agronomic and quality characters has provided the possibility of introgressing desirable characters among gene pools while minimizing the effects of linkage drag of undesirable loci associated with the target traits. These observations once again demonstrate the opportunities offered by MAS to make breeding programs more efficient and responsive

## Supporting Information

S1 Table Map and markers data of Norstar by Cappelle Desprez. (XLSX)

S2 Table Map and markers data of Norstar by Manitou. (XLSX)

S3 Table Map and markers data of Norstar by winter Manitou. (XLSX)
